# Nonconsumptive predator effects modify crayfish‐induced bioturbation as mediated by limb loss: Field and mesocosm experiments

**DOI:** 10.1002/ece3.5444

**Published:** 2019-09-30

**Authors:** Luc A. Dunoyer, Dakota Coomes, Philip H. Crowley

**Affiliations:** ^1^ Department of Biology University of Kentucky Lexington KY USA

**Keywords:** autotomy, ecosystem engineering, enclosure‐exclosure experiments, *Faxonius rusticus*, turbidity

## Abstract

1. We addressed the implications of limb loss and regeneration for multispecies interactions and their impacts on ecosystem engineering in freshwater stream environments.
2. We included regenerative and nonregenerative crayfish as well as fish predators in a 2 × 2 factorial design to assess the effects on water turbidity of interactions between crayfish ecosystem engineers differing in regenerative status and their fish predators.
3. We demonstrated that crayfish limb loss and predation risks lead to more turbidity in field and mesocosm conditions. Moreover, ongoing regeneration of crayfish increased turbidity, while fish presence seemed to hinder crayfish turbidity‐inducing behaviors (such as tail‐flipping and burrowing) in the mesocosm experiment.
4. We confirmed that greater numbers of crayfish produce a greater amount of turbidity in situ in streams.
5. Although mechanical burrowing crayfish capacities may depend on crayfish burrowing classification (primary, secondary, or tertiary), our work emphasizes the implication for turbidity levels of crayfish autotomy in freshwater streams.

## Graphical abstract

We addressed the implications of limb loss and regeneration for multispecies interactions and their impacts on ecosystem engineering in freshwater stream environments. We demonstrated that crayfish limb loss and predation risks lead to more turbidity in field and mesocosm conditions. Although mechanical burrowing crayfish capacities may depend on crayfish burrowing classification (primary, secondary, or tertiary), our work emphasizes the implication for turbidity levels of crayfish autotomy in freshwater streams.

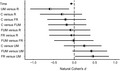

## 1 INTRODUCTION

Ecosystem engineering is the modification of the physical environment from one state to another by organisms (Jones Lawton, & Shachak, 1994, 1997b). Almost every organism on Earth engineers its environment to some degree (Jones, Lawton, & Shachak, 1997a). The importance of ecosystem engineering in each case depends on its intensity and potential for cascading effects (similar to trophic interactions; Wilby, 2002). The effects of ecosystem engineering have been extensively investigated, including biodiversity effects (Caliman et al., 2013), management options (Byers et al., 2006), and implications for ecosystem services (Daily, 1997). However, little is known about other ecological and physiological processes may influence the effect of ecosystem engineering within the ecosystem (Folgarait, 1998; Rietkerk, Dekker, Ruiter, & Koppel, 2004). Environmental impacts of ecosystem engineers mediated by nonconsumptive effects of the engineers' predators are one of those interactions. Previous studies have focused on density‐mediated interaction (Nishijima, Takimoto, & Miyashita, 2016; Sanders et al., 2014; Sanders & van Veen, 2011; Wilby, Shachak, & Boeken, 2001), whereas we focus here on trait‐mediated interaction.

We develop a case study of such effects by looking at the nonconsumptive impact of fish on crayfish engineering in freshwater streams, combining in situ and ex situ experimental approaches. Organic resource availability in stream communities contributes to species biodiversity (Vannote, Minshall, Cummins, Sedell, & Cushing, 1980) and ultimately to ecosystem services, though often in complex ways. These resources are influenced by bioturbation, a form of ecosystem engineering based on increased turbidity in an aquatic system resulting from biological activity (Meysman, Middelburg, & Heip, 2006). Bioturbation is known to diminish light penetration in the water column, leading to a reduction of primary production in marine and freshwater systems (Ciutat, Anschutz, Gerino, & Boudou, 2005; Fager, 1964; Heinzelmann & Wallisch, 1991; Mermillod‐Blondin & Rosenberg, 2006). Previous research has explored this process by investigating single macroinvertebrate taxa in isolation, most commonly crayfish (Creed & Reed, 2004) or microinvertebrates (Duarte, Fidalgo, Pascoal, & Cassio, 2012). To our knowledge, however, the effects of interactions between taxa have not been shown to influence bioturbation (Usio & Townsend, 2001).

Crayfish are well‐known ecosystem engineers that greatly influence the availability of organic resources directly by shredding leaf litter and consuming microinvertebrates (Dunoyer, Dijoux, Bollache, & Lagrue, 2014; Momot, 1995) or indirectly via bioturbation during burrow construction or sediment displacement (Angeler, Sánchez‐Carrillo, García, & Alvarez‐Cobelas, 2001; Usio & Townsend, 2004; Yamamoto, 2010). Crayfish induce bioturbation simply by walking on the substrate (Statzner & Sagnes, 2008), anchoring themselves to the stream bed in fast flowing current areas (Maude & Williams, 1983), tail‐flipping when evading predators, and especially by burrowing (Statzner, 2012). These behaviors result in long‐term effects on bed stream composition (Statzner, 2012).

Like most arthropods, crayfish are capable of regeneration following the loss of a limb or other appendages via autotomy (Wood & Wood, 1932). Large fish, birds, raccoons, and other predators of crayfish can have direct impacts on crayfish through predation and indirect nonlethal effects by inducing limb loss. Natural populations of crustaceans and crayfish have up to 30% of individuals regenerating a missing limb at any given time (Juanes & Smith, 1995; Kouba, Buřič, Policar, & Kozák, 2011; Powell, Stephen, & Watts, 1998). Because regeneration is a slow and costly process that inhibits burrow construction (one clawed crayfish are incapable of burrowing; L. A. Dunoyer, unpublished data), predator‐induced injuries substantially influence bioturbation at least until regeneration is completed and burrowing capacities are fully recovered (L. A. Dunoyer, unpublished data).

Crayfish‐induced turbidity (Maude & Williams, 1983; Statzner & Sagnes, 2008) reduces light penetration and primary production in wetland habitat characterized by low water flow (Anastacio, Correia, Menino, & Silva, 2005). This reduction in primary production can in turn affect diversity in those environments (Rodríguez, Bécares, Fernández‐Aláez, & Fernández‐Aláez, 2005). Turbidity also changes fish predator avoidance by crayfish. The skew of mortality pattern toward small individuals in clear water is eliminated in turbid water (Abrahams & Kattenfeld, 1997; Kimbell & Morrell, 2016). Furthermore, fish are less able to escape rapid attacks from other fish in turbid water, while the opposite is true when facing a slow predator (Meager, Domenici, Shingles, & Utne‐Palm, 2006). Those studies underscore the importance of water turbidity in shaping habitat uses and predation patterns by fish. For crayfish, conspecific chemical alarm cues are more important than odor cues from fish for predator avoidance (Gherardi, Mavuti, Pacini, Tricarico, & Harper, 2011). Regenerative crayfish may induce higher turbidity through inefficient burrowing (since crayfish rely on their cheliped to burrow; pers. comm.; Berrill & Chenoweth, 1982; Helms et al., 2013) and thereby impede detection by predators.

We aim to assess the consequences of nonconsumptive predator effects on crayfish‐induced bioturbation. First, we predict that regenerating crayfish will have an increased bioturbidity impact compared to their unmanipulated counterparts due to their reduced burrowing capacity (they will struggle more to accomplish similar burrowing output). Second, if predators influence this ecosystem engineering process, we predict that crayfish‐induced bioturbation will be further increased in the presence of a predatory fish, in an attempt to provide more protection against predation.

## 2 METHODS

### 2.1 Study sites

Our field experiment was conducted in the Green River drainage of the Ohio River watershed in Kentucky, USA (Subregion Hydrologic Unit Code 4‐digit: 0511). This area is located at the convergence of the Cincinnati Arch with the Appalachian Basin, resulting in a highly diverse assemblage of freshwater species (103 of the 297 species of North American mussel species, 248 species of freshwater fish, 57 amphibian species, and 54 out of the 360 North American crayfish species; Kentucky Department of Fish & Wildlife Resources, 2013). We used three stream sites in nearby but separate creeks for our experiments (GPS coordinates): site 1 (37.333699, −85.420170); site 2 (37.342768, −85.458752); site 3 (37.384984, −85.463014).

In addition to our field experiment, we also conducted a mesocosm experiment using artificial pools outside at a field station to determine the specific effects of predation by fish (and subsequent regeneration by crayfish) on the crayfish bioturbation process. Mesocosm experiments allow us more control of environmental variation. The site was the University of Kentucky's Ecological Research and Education Center (EREC) field station in Lexington, Kentucky. The same completely randomized design was used for a single block (see below), with fifteen 40 gallon plastic tanks placed under shade cloth mesh and half dug into the ground to mimic stream conditions in slow‐moving water flow under canopy. The substrate in each tank was a mix of gravel and sand similar to what was found in the streams in the field.

### 2.2 Study species

We used kick sampling (Mather & Stein, 1993) to capture native crayfish (*Faxonius rusticus*, Girard 1852; Crandall & De Grave, 2017) and nonlethal electro‐fishing methodology (Cowx & Lamarque, 1990) to capture crayfish predators (fish—rock bass, *Ambloplites rupestris*, a known predator of crayfish), then used the fish and crayfish to establish a predator enclosure–exclosure experiment in the field. Before addition to the enclosures, crayfish were measured from the back of the orbit to the center of the dorso‐posterior margin of the carapace (within 0.1 mm, OCL; size range = 3.5–9 cm; average size ± standard deviation = 5.56 ± 1.27 cm). Fish were measured from the tip of the snout to the tip of the longer lobe of the caudal fin (Total Length, TL; size range = 7–12 cm; average size ± standard deviation = 9.83 cm ± 1.34 cm). All fish behaved normally in the enclosures and were all healthy at the end of the experiment.

### 2.3 Experimental design and methods

**Figure 1:**
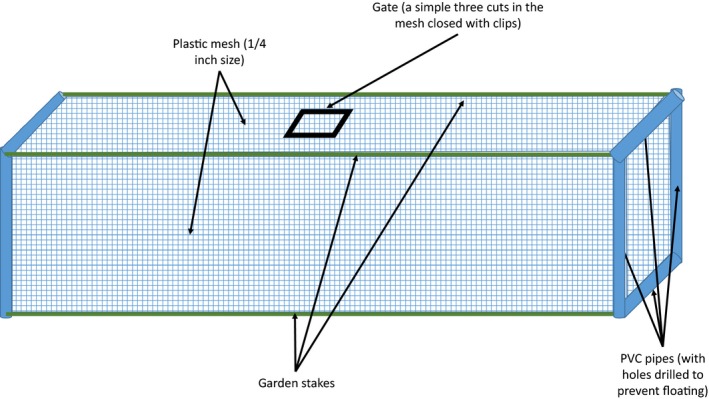
Diagram representing the enclosure–exclosure design

Each enclosure–exclosure was a 3D rectangle of 90 × 30 × 30 cm made out of a frame of PVC pipes (drilled to prevent floating) and garden stakes (Figure 1). The structure was then covered with 0.6 cm plastic mesh attached with zip locks. Finally, a door was made atop the structure in the plastic mesh to allow addition of the animals.

The study design was a randomized complete block, with three replicates of five treatments within each stream location for each of the three remaining stream locations. Each enclosure–exclosure in each block of 15 was placed at least five meters from each other and never directly downstream from one another (staggered placement); thus, each replicate and treatment was not influenced by other replicates and treatments. Treatments were as follows: (a) fish and crayfish excluded—control (C); (b) fish excluded, unmanipulated crayfish added (UM); (c) fish excluded, regenerating crayfish (limb autotomized) added (R); (d) fish added, unmanipulated crayfish added (FUM); and (e) fish added, regenerating crayfish added (FR). Treatments were added into the enclosure–exclosure placed in a slow water flow area. We shoveled 5 cm of substrate over the bottom surface of each, effectively sealing them to the streambed by embedding the bottom mesh into the sediment.

We quantified the crayfish bioturbation process by sampling turbidity (in NTU using a LaMotte 2020we Turbidity Meter) directly downstream of the enclosure–exclosure channels (to prevent the influence of nearby/upstream outside factors) every week, starting a week after setting up the experiment. The turbidity meter processed a small water sample at the field site. Moreover, we gathered water samples from the downstream end of the enclosures to prevent influences on subsequent measurements. This experiment ran from September 12 to October 13, 2015. Both field and mesocosm experiments lasted four weeks, yielding 48 temporal data points per treatment (4 locations (3 field sites and 1 mesocosms site) * 3 replicates * 4 weeks). We also assessed how body size affects regeneration and bioturbation processes. Fish slightly larger than the crayfish were chosen when paired in the enclosure‐exclosure in the field experiment or in the artificial pools in the mesocosms experiment. This size pairing included fish large enough to be perceived as a predation threat by crayfish, while not large enough to actually consume the crayfish. Finally, neither fish nor crayfish were expected to grow significantly during the experiment duration.

### 2.4 Data analyses

All analyses were conducted in R (R Core Team, 2017) using several additional packages (ggplot2, Wickham, 2009; cowplot, Wilke, 2017; nlme, Pinheiro, Bates, DebRoy, & Sarkar, 2017; sjstats, Lüdecke, 2018; MuMin, Barton, 2017; dplyr, Wickham, Francois, Henry, & Müller, 2017; gridExtra, Anguie, 2017; psych, Revelle, 2017; car, Fox & Weisberg, 2011).

Field and mesocosm data were analyzed separately using information theory (Burnham & Anderson, 2002). This approach ranks several models in a set and allows multimodel inferences using evidence of statistical support from the given dataset based on each model fit. First, we determined the best fit for our complete mixed effects model, addressing our hypothesis about the consequences of nonconsumptive predator effects on crayfish‐induced bioturbation in stream and pond, respectively. The dependent variable was turbidity‐induced (in NTU) following a normal distribution. The different variables used in the mixed effects models were time, treatment (see above), their interaction, and fish as well as crayfish length at the start of the experiment (TL and OCL, respectively). Site was chosen as a random factor for the field experiment to allow for unknown differences between sites influencing turbidity measurements. Furthermore, a specific variance structure was incorporated to improve model fit as assessed graphically. This structure accounts for the variance of the covariate crayfish length per treatment level while allowing for each stratum of fish length to have different variances (Zuur, Ieno, Walker, Saveliev, & Smith, 2009).

Subsequently, based on our complete model, we compared all nested models and determined the best models from this set using information theory (see above). All models derived from the parent model were compared using the corrected Akaike Information Criterion (AIC_c_) and Akaike weights (*ω_i_*; Anderson & Burnham, 2002; Anderson, Link, Johnson, & Burnham, 2001; Burnham, Anderson, & Huyvaert, 2011; Table 1). AICc represents model fit with smaller values being better fits, and the best‐supported models include all models with ΔAIC_c_ < 2 (Burnham & Anderson, 2002). Akaike weights represent strength of evidence for each model in a given data set (Burnham & Anderson, 2002). Additionally, alternate Akaike weights were computed for each variable to assess their individual importance (as the sum of the ω_i_ of the models in which the variable is present). Although the Akaike weights of all models add up to 1, alternate Akaike weights for individual variables do not generally sum to 1; these individual‐variable sums are between 0 and 1, where 0 denotes a lack of importance and 1 a high importance of the considered variable in the model set (Burnham & Anderson, 2002). Finally, Cohen's *d* values were computed to report the effect sizes associated with variables having large Akaike weights. These effect sizes were calculated on nonstandardized data for repeatability and comparability of our results with future research (Bakeman, 2005; Morris & DeShon, 2002). However, unstandardized effect sizes ignore the structure of the data set (i.e., here repeated measurements and stream identities). Hence, we consider all effect sizes with confidence intervals not at all or slightly overlapping the null value as well as effect sizes from relevant comparisons (involving controls for example). In doing so, we address both the amount of overlap between effect size confidence intervals and zero and the magnitudes of the effect sizes themselves. By presenting the entire set of effect sizes with their confidence intervals (for the treatment covariate), we are allowing readers to interpret the results for themselves and reach their own conclusions, while providing our own interpretation, hoping that these two are ultimately in agreement.

**Table 1:** Information theory output of the field models

Model^a^	Treatment^c^	Date^d^	Crayfish length^e^	Fish length^f^	Treatment × Date^g^	*df*	AIC_c_	ΔAIC_c_	*ω_i_* (%)
I^b^		−0.06803				16	890.9		32.70
G	+	−0.06923				20	891.3	0.39	26.90
F	+	−0.06865	−0.3021			21	891.8	0.94	20.40
H	+					19	894.0	3.08	7.00
E	+	−0.06965		−0.005515		21	894.1	3.16	6.60
B	+	0.06923	−0.3014	0.039080		22	894.7	3.82	4.80
D	+		−0.3007	−0.125000		21	897.5	6.61	1.20
C		−0.06747	−0.1836	0.048820		18	900.1	9.16	0.30
A	+	−0.09152	−0.03030	−0.042720	+	26	920.5	29.57	0.00

a A particular model (row) contained a particular variable either if there is a “+” (categorical variable) or if there is a coefficient (continuous variable) in the respective variable column. Site was chosen as a random factor to control for any unmeasured differences between sites impacting our turbidity measurements. Finally, a variance structure was implemented to improve model fit (following crayfish length per treatment level and fish length, see Section 2).

b For example, the model I is Turbidity ~ Date.

c Treatment variable Akaike weight = 0.67 (appeared in seven models).

d Date variable Akaike weight = 0.92 (appeared in seven models).

e Crayfish length variable Akaike weight = 0.27 (appeared in five models).

f Fish length variable Akaike weight = 0.13 (appeared in five models).

g Treatment and Date interaction variable Akaike weight < 0.01 (appeared in one model).

Finally and a posteriori, we took advantage of the whole data set by analyzing the data using the last data recorded at each stream. We regressed the final number of crayfish found both per channel and per stream against the final turbidity measurement per channel and per stream (averaged), respectively.

Our study protocol and procedures were ethically reviewed and approved by the Institutional Animal Care and Use Committee at the University of Kentucky (protocol #2015‐2068). All the data and R script with packages used in this work are available on Dryad (https://doi.org/10.5061/dryad.58k2h35).

## 3 RESULTS

### 3.1 Consequences of nonconsumptive predator effects on crayfish‐induced bioturbation in the field experiment

**Figure 2:**
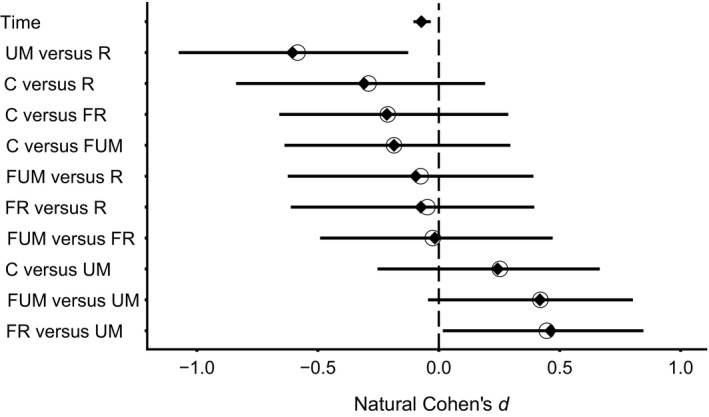
Effect sizes associated with the variables “Treatment” and “Date” in the field part of the experiment. The diamonds and lines are the average Cohen's *d* values with 95% confidence intervals after 10,000 bootstraps except for the variable “Date” for which the mean value is simply the average of its coefficients in all the considered models (see Table 1). Open circles represent the Cohen's *d* value calculated on the experimental data rather than from the 10,000 bootstraps. A variable has a significant effect on turbidity if its 95% confidence interval does not overlap with 0 (the dashed line). C, control; FR, fish with regenerating crayfish; FUM, fish with unmanipulated crayfish; R, regenerating crayfish; UM, unmanipulated crayfish

**Figure 3:**
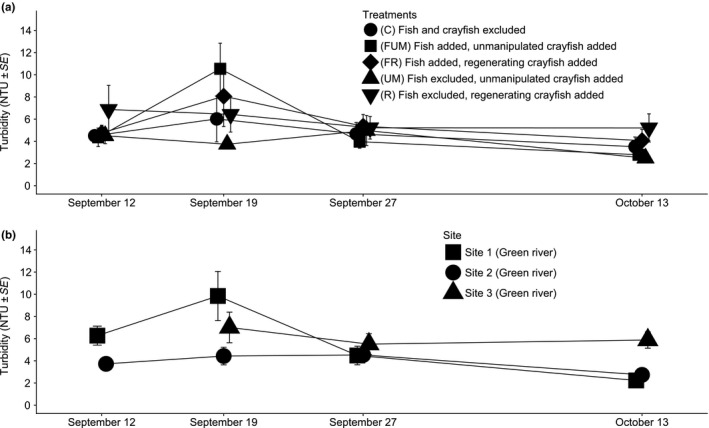
The evolution of turbidity over time in the field experiment. (a) Breakdown of turbidity by treatment. (b) Breakdown of turbidity by field site. See Section 2 for details

The best‐supported models identified from the set of nested models using the Information Theory approach were models “I” (Turbidity ~ Date), “G” (Turbidity ~ Treatment + Date), and “F” (Turbidity ~ Treatment + Date + Crayfish Length; Table 1). Among the set of models run through the Information Theory approach, neither fish nor crayfish size affected turbidity; likewise, the interaction between time since the start of the experiment and treatments did not affect turbidity (Table 1). Turbidity decreased with time since the start of the experiment (Coefficient_time_ = −0.07, CI95%_time_ = [−0.10; −0.03]; Figure 2). Since turbidity over time does not seem to affect control and treatment condition differently (nonsignificant interaction; Figure 3), this impact is likely due to the stream background turbidity. Furthermore, the treatment covariate significantly influenced turbidity; specifically, unmanipulated crayfish treatment created less turbidity than regenerating crayfish or fish with regenerating crayfish treatments (Cohen's *d*_UMvsR_ = −0.60, CI95%_UMvsR_ = [−1.08; −0.13]; Cohen's *d*_FRvsUM_ = 0.46, CI95%_FRvsUM_ = [0.01; 0.87]; Figure 2; Table 1). Furthermore, there was a nonsignificant trend toward higher turbidity for treatments with fish and unmanipulated crayfish than for those with unmanipulated crayfish alone (Cohen's *d*_FUMvsUM_ = 0.42, CI95%_FUMvsUM_ = [−0.03; 0.81]; Figure 2; Table 1). Finally, while statistically undistinguishable from controls, each treatment condition except unmanipulated crayfish induced more turbidity than the control treatment (Cohen's *d*_CvsR_ = −0.32, CI95%_CvsR_ = [−0.86; 0.18]; Cohen's *d*_CvsFR_ = −0.21, CI95%_CvsFR_ = [−0.67; 0.28]; Cohen's *d*_CvsFUM_ = −0.19, CI95%_CvsFUM_ = [−0.65; 0.32]; Cohen's *d*_CvsUM_ = 0.25, CI95%_CvsUM_ = [−0.24; 0.68]; Figure 2; Table 1). Overall, in the field experiment, regenerating crayfish induced more turbidity than their unmanipulated counterparts while predatory fish presence always enhanced turbidity.

### 3.2 Consequences of nonconsumptive predator effects on crayfish‐induced bioturbation in the Mesocosm experiment

**Figure 4:**
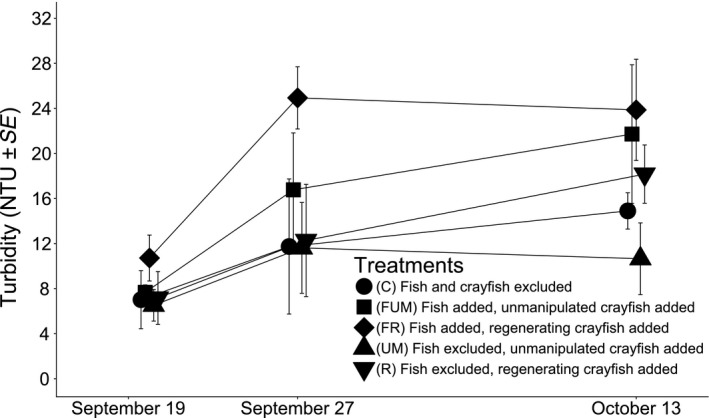
The evolution of turbidity over time in the mesocosms experiment with a breakdown of turbidity by treatment. See Section 22 for details

**Figure 5:**
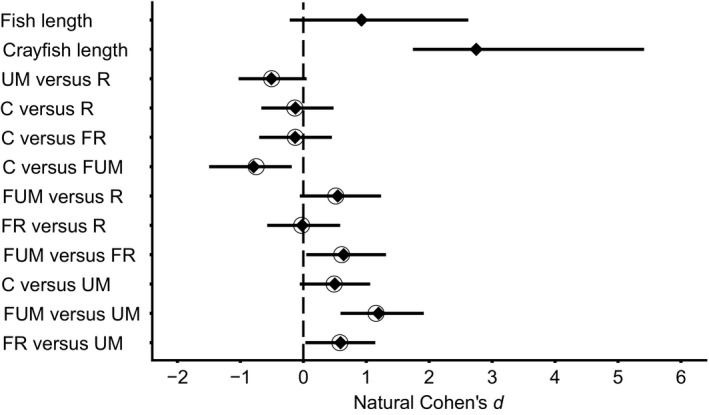
Effect sizes associated with the variables “Treatment,” “Crayfish length,” and “Fish length” in the mesocosms part of the experiment. The diamonds and lines are the average Cohen's *d* values with 95% confidence intervals after 10,000 bootstraps except for the variables “Crayfish length” and “Fish length,” for which the mean values are simply the average of their coefficients in all the considered models (see Table 2). Open circles represent the Cohen's *d* value calculated on the experimental data rather than from the 10,000 bootstraps. A variable has a significant effect on turbidity if its 95% confidence interval does not overlap with 0 (the dashed line). C, control; FUM, fish with unmanipulated crayfish; FR, fish with regenerating crayfish; R, regenerating crayfish; UM, unmanipulated crayfish. The treatment factor contrasts have been ordered similarly to Figure 1 to facilitate visual comparison

The best‐supported models identified from the set of nested models using the Information Theory approach was model “D” (Turbidity ~ Treatment +Date + Crayfish Length + Fish Length; Table 2). Neither time since the start of the experiment nor its interaction with treatments affected turbidity (Figure 4); however, crayfish and fish sizes significantly increased turbidity, while the treatment covariate also significantly influenced turbidity (Coefficient_crayfish_length_ = 2.75, CI95%_crayfish_length_ = [1.74; 5.41]; Coefficient_fish_length_ = 0.92, CI95%_fish_length_ = [−0.21; 2.62]; *ω*_treatment_ = 1; Figure 5; Table 2). Specifically, fish with unmanipulated crayfish treatment created more turbidity than controls or fish with regenerating crayfish or unmanipulated crayfish treatments, while unmanipulated crayfish treatment created less turbidity than fish with regenerating crayfish treatment (Cohen's *d*_CvsFUM_ = −0.79, CI95%_CvsFUM_ = [−1.49; −0.19]; Cohen's *d*_FUMvsFR_ = 0.64; CI95%_FUMvsFR_ = [0.05; 1.30]; Cohen's *d*_FUMvsUM_ = 1.21; CI95%_FUMvsUM_ = [0.59; 1.93]; Cohen's *d*_FRvsUM_ = 0.59; CI95%_FRvsUM_ = [0.04; 1.14]; Figure 5; Table 2). Finally, there was a nonsignificant trend suggesting that unmanipulated crayfish tend to induce less turbidity than regenerating crayfish or control, while fish with unmanipulated crayfish tended toward inducing more turbidity than regenerating crayfish (Cohen's *d*_UMvsR_ = −0.50, CI95%_UMvsR_ = [−1.03; 0.05]; Cohen's *d*_CvsUM_ = 0.50, CI95%_CvsUM_ = [−0.06; 1.06]; Cohen's *d*_FUMvsR_ = 0.55, CI95%_FUMvsR_ = [−0.05; 1.23]; Figure 5; Table 2). Overall, in the mesocosm experiment, once again predatory fish presence always increased turbidity, while regenerating crayfish induced more turbidity than their unmanipulated counterparts. However, this last result did not hold true when regenerating crayfish were paired with a predatory fish, seemingly hindering regenerating crayfish‐induced turbidity in the mesocosm experiment.

**Table 2:** Information theory output of the mesocosms models

Model^a^	Treatment^c^	Date^d^	Crayfish length^e^	Fish length^f^	Treatment × Date^g^	*df*	AIC_c_	ΔAIC_c_	*ω_i_* (%)
D^b^	+		3.5830	1.2030		15	810.0		96.3
B	+	0.01103	3.6050	1.2020		16	818.3	8.22	1.6
H	+					13	818.8	8.78	1.2
F	+	0.01245	2.9690			15	819.3	9.25	0.9
G	+	0.01104				14	826.9	16.87	0
E	+	0.01134		0.3245		15	828.1	18.08	0
A	+	0.10160	3.8730	1.2670	+	20	831.1	21.09	0
C		0.01006	−0.2935	0.6230		12	844.3	34.22	0
I		0.01363				10	849.7	39.66	0

a A particular model (row) contained a particular variable either if there is a “+” (categorical variable) or if there is a coefficient (continuous variable) in the respective variable column. Site was chosen as a random factor to control for any unmeasured differences between sites impacting our turbidity measurements. Finally, a variance structure was implemented to improve model fit (following crayfish length per treatment level and fish length, see Section 2).

b For example, the model D is Turbidity ~ Treatment +Crayfish length + Fish length.

c Treatment variable Akaike weight = 1 (appeared in seven models).

d Date variable Akaike weight = 0.03 (appeared in seven models).

e Crayfish length variable Akaike weight = 0.99 (appeared in five models).

f Fish length variable Akaike weight = 0.98 (appeared in five models).

g Treatment and Date interaction variable Akaike weight < 0.01 (appeared in one model).

### 3.3 Testing the impact of crayfish on turbidity—an a posteriori analysis

**Figure 6:**
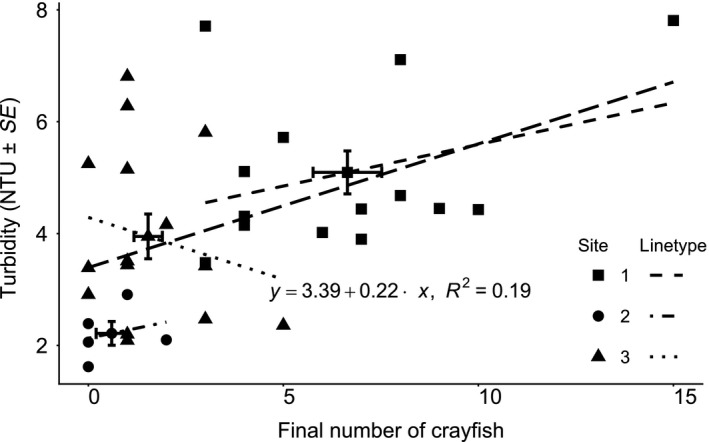
A posteriori regressions. Scatter plots of the numbers of crayfish against turbidity at the end of the experiment in each remaining channels with site averages. The long‐dash regression line, equation, and *R* ^2^ value correspond to channels' turbidity and not site averaged turbidity (see text for details about each regression per stream). Note that the analysis has already gone through outlier assessment procedures. Finally, if the seemingly extreme data point (*x* = 15, *y* ~ 8) is removed, model fit is greatly impeded as assessed graphically (Normal *Q*–*Q*, Residuals vs. Fitted, Scale‐Location, Residual vs. Leverage, and cook's distances plots)

We found a positive relationship between the turbidity and the number of crayfish at the end of the experiment in each channel (*m* = 0.22; *df* = 32; *F*‐statistic = 8.802; *p*‐value = 0.006; *R* ^2^_adj._ = 0.19; Figure 6). However, we found no relationship between the turbidity and the number of crayfish at the end of the experiment per stream (*m* = 0.39; *df* = 1; *F*‐statistic = 3.393; *p*‐value = 0.32; Figure 6). This lack of significance, despite a positive trend (*R* ^2^_adj._ = 0.55), may reflect the relatively small sample size. Finally, we found no relationships between the turbidity and the number of crayfish at the end of the experiment at each individual stream (Site 3: *m* = 0.15, *df* = 12, *F*‐statistic = 1.582, *p*‐value = 0.23; Site 4: *m* = 0.14, *df* = 3, *F*‐statistic = 0.2387, *p*‐value = 0.66; Site 5: *m* = −0.22, *df* = 13, *F*‐statistic = 0.5498, *p*‐value = 0.47; Figure 6). Overall, more crayfish induced more turbidity at the relevant scale in the field experiment.

## 4 DISCUSSION

Our results demonstrated that greater crayfish abundance in a location induces higher turbidity at a small spatial scale (between channels irrespective of location) compared to larger scale (between streams). We also showed that crayfish limb loss and predation risk lead to more turbidity in field and mesocosm conditions. Furthermore, larger crayfish induce more turbidity than smaller crayfish under mesocosm conditions. Finally, experimental removal of a crayfish chela did increase turbidity. However, we did not find an effect of fish size on turbidity in the field or mesocosm experiments. Nonetheless, fish presence seems to hinder crayfish turbidity‐inducing behaviors in the mesocosm experiment, despite an overall turbidity increase. The direct influence of crayfish number and size on turbidity confirms the importance of crayfish as turbidity‐inducing organisms in freshwater streams. Nonetheless, several results were unexpected and deserve closer attention.

Crayfish burrowing behaviors have been linked to increased soil respiration (Richardson, 1983) and phosphorus mixing in soil (Stone, 1993). In freshwater systems, fish and crayfish have been shown to increase bioturbation (Tables 1 and 2; Statzner, 2012). Here we provided a case study of the interactive effect of fish and crayfish on crayfish bioturbation in streams. Our study suggests that predation avoidance by crayfish increased turbidity when predatory fish were present. This turbidity can be induced either by walking on the streambed (Statzner, 2012), tail‐flipping, or burrowing for shelter (Dorn & Mittelbach, 1999; Ilheu, Acquistapace, Benvenuto, & Gherardi, 2003).

We also demonstrated that limb loss can enhance crayfish‐induced turbidity, which may account for a substantial proportion of natural turbidity. Crayfish use their two chelipeds to excavate and mold mud pellets during burrowing (Berrill & Chenoweth, 1982; Helms et al., 2013; L. A. Dunoyer, personal observations), often forming chimneys on top of their burrows. One clawed crayfish can only burrow a depression in the ground rather than a functioning burrow (L. A. Dunoyer, unpublished data). Nonetheless, regenerating crayfish may avoid exposure as much as possible while undergoing limb regeneration, having only one fully functional cheliped for protection from predators. Since burrowing is inefficient for autotomized crayfish, they avoid predation in turbidity‐enhancing ways by walking on the streambed and/or tail‐flipping when evading predators (Statzner, 2012). Moreover, when trying to burrow for shelter, autotomized crayfish are less efficient than their unmanipulated counterparts, generating more turbidity in the process.

Intrinsic differences between experimental streams and mesocosms could have caused the lack of increased turbidity for regenerating crayfish when paired with predatory fish. Flowing water might rapidly dilute and remove chemical cues of fish predators. Thus, crayfish in streams might have been less aware of fish presence than their conspecifics in the mesocosm experiment, where predatory may last longer. This reduced predator awareness could have allowed exploratory and burrowing behavior to continue in the presence of a predator, resulting in greater turbidity in stream environments. Alternatively, the control treatment in the field might have included exposure to predator cues, unlike the control treatments in the mesocosm experiment. It is likely that only fish were generating turbidity when paired with regenerating crayfish in the mesocosms, since autotomize crayfish must reduce exploratory and burrowing behavior to avoid predation. In contrast, control crayfish possessing both chelipeds were less likely to adjust their behavior, having both double cheliped defense and cheliped autotomy available in the presence of the predator.

Increased energy requirements due to regeneration following autotomy may also contribute to increased foraging activity by regenerating crayfish. However, this explanation relies on two assumptions empirically unsupported. First, limb loss and regeneration are assumed to induce an increase in crayfish energy budget. Second, if indeed limb loss and regeneration do induce an increased energy requirement, crayfish are then assumed to fulfill this increase by foraging more. Thus, increased foraging implies more movement on the stream bed leading to more turbidity (Statzner, 2012). Alternatively, crayfish might be able to regenerate using energy reserves or otherwise adjust energy utilization without the need to increase their energy uptake.

Alternatively, crayfish‐induced turbidity following limb loss may be attributed to predation avoidance. Indeed, fish predation efficiency is reduced in turbid water (Abrahams & Kattenfeld, 1997; Kimbell & Morrell, 2016; Meager et al., 2006). However, it is unclear if the observed range of turbidity in this experiment (between 0 and 8 NTU) affects crayfish predation by fish. Studies of fish predation on crayfish at different turbidity levels are needed to resolve this.

Our work emphasizes the role of crayfish behavior and autotomy, depending on the level of predation risk, in determining turbidity levels in freshwater streams. Because species live in a community context and by uncovering the complexity of crayfish–fish interactions, we raised questions about the ways that crayfish induce turbidity as well as about the explanation for crayfish behavioral changes following cheliped autotomy. It is our hope that future research will uncover both mechanisms and causes of crayfish behavioral change induced by autotomy.

## CONFLICT OF INTERESTS

The authors declare no potential sources of conflict of interest.

## AUTHOR CONTRIBUTIONS

LAD designed the experiment. DC and LAD conducted the experiment. DC was an undergraduate in the Crowley laboratory under LAD's direct supervision. LAD conducted the statistical analyses and wrote the first draft of this manuscript. LAD and PHC shaped the manuscript into final form.

## DATA ACCESSIBILITY

R script, packages, and data are available on Dryad (https://doi.org/10.5061/dryad.58k2h35).
